# Cassava brown streak disease in Rwanda, the associated viruses and disease phenotypes

**DOI:** 10.1111/ppa.12789

**Published:** 2017-11-09

**Authors:** E. Munganyinka, E. M. Ateka, A. W. Kihurani, M. C. Kanyange, F. Tairo, P. Sseruwagi, J. Ndunguru

**Affiliations:** ^1^ Rwanda Agriculture Board PO Box 5016 Kigali Rwanda; ^2^ Jomo Kenyatta University of Agriculture and Technology PO Box 62000‐00200 Nairobi Kenya; ^3^ Mikocheni Agricultural Research Institute PO Box 6226 Dar es Salaam Tanzania; ^4^ Karatina University PO Box 1957‐10101 Karatina Kenya

**Keywords:** cassava, cassava brown streak disease, phylogenetic analysis, RT‐PCR, symptom expression

## Abstract

Cassava brown streak disease (CBSD) was first observed on cassava (*Manihot esculenta*) in Rwanda in 2009. In 2014 eight major cassava‐growing districts in the country were surveyed to determine the distribution and variability of symptom phenotypes associated with CBSD, and the genetic diversity of cassava brown streak viruses. Distribution of the CBSD symptom phenotypes and their combinations varied greatly between districts, cultivars and their associated viruses. The symptoms on leaf alone recorded the highest (32.2%) incidence, followed by roots (25.7%), leaf + stem (20.3%), leaf + root (10.4%), leaf + stem + root (5.2%), stem + root (3.7%), and stem (2.5%) symptoms. Analysis by RT‐PCR showed that single infections of *Ugandan cassava brown streak virus* (UCBSV) were most common (74.2% of total infections) and associated with all the seven phenotypes studied. Single infections of *Cassava brown streak virus* (CBSV) were predominant (15.3% of total infections) in CBSD‐affected plants showing symptoms on stems alone. Mixed infections (CBSV + UCBSV) comprised 10.5% of total infections and predominated in the combinations of leaf + stem + root phenotypes. Phylogenetic analysis and the estimates of evolutionary divergence, using partial sequences (210 nt) of the coat protein gene, revealed that in Rwanda there is one type of CBSV and an indication of diverse UCBSV. This study is the first to report the occurrence and distribution of both CBSV and UCBSV based on molecular techniques in Rwanda.

## Introduction

Cassava (*Manihot esculenta*) greatly enhances food security in the tropics and subtropics. In Rwanda, it ranks third to bananas and sweet potatoes as a staple crop, with an annual production of approximately 402 436 × 10^6^ tonnes (Rwandan Ministry of Agriculture and Animal Resources (MINAGRI), 2011). The crop is grown mainly in the southern and eastern provinces of the country and occupies 21.5% of the area under cultivation. More than 700 000 households (i.e. 42% of all family farms) grow cassava on an annual basis (MINAGRI, 2011). The crop is a new cash crop and can be sold as fresh roots, dry chips, flour and flour products; therefore, it is a good source of income for many rural households.

Cassava production in Africa is hampered by two viral diseases: cassava mosaic disease (CMD), caused by cassava mosaic geminiviruses, and cassava brown streak disease (CBSD), caused by cassava brown streak viruses (Legg & Raya, 1998; Hillocks & Thresh, 2000). CBSD poses a serious threat because infected roots cannot be consumed (Gondwe *et al*., 2003). Considerable variation in isolates of cassava brown streak viruses has been reported (Monger *et al*., 2001; Winter *et al*., 2010), leading to the confirmation of the existence of two distinct viral species: *Cassava brown streak virus* (CBSV) and *Ugandan cassava brown streak virus* (UCBSV; Mbanzibwa *et al*., 2009). Both viruses belong to the genus *Ipomovirus*, family *Potyviridae* (Monger *et al*., 2001). Studies on characterization and evolution of cassava brown streak viruses have revealed different genetic sequences between CBSV and UCBSV, and that genetic diversity is wider among CBSV isolates (Monger *et al*., 2010; Mbanzibwa *et al*., 2011a). A recent report suggests that there may be as many as four distinct viral species causing CBSD (Ndunguru *et al*., 2015; Alicai *et al*., 2016).

Cassava brown streak viruses are transmitted by an insect vector, *Bemisia tabaci* (Maruthi *et al*., 2005), by graft inoculation (Storey, 1936) and by mechanical inoculation in herbaceous host plants (Mbanzibwa *et al*., 2009; Ogwok *et al*., 2010). Similarly, the use of infected cassava cuttings spreads the virus in the field. Infected plants produce low yields of poor quality. CBSD presents a variable pattern of symptoms, which makes symptom‐based diagnosis difficult. Aerial symptoms are seen predominantly as leaf chlorosis in feathery patterns along the margins of tertiary veins that may later develop into chlorotic blotches. Infected stems present with brown lesions or streaks, resulting in stem dieback in severe infections (Jennings, 1960; Winter *et al*., 2010). Root symptoms are characterized by a reduction in root size and the formation of radial constrictions and necrotic lesions within the root (Hillocks & Thresh, 2000). Symptoms associated with CBSD vary with crop variety, age at time of infection and the prevailing environmental conditions (Legg & Thresh, 2003).

The first report of CBSD was made at the foothills of the Usumbara mountains in present‐day Tanzania (Storey, 1936). Later studies showed that the disease was present in all cassava‐growing areas of the East African Coast, from the northeast border of Kenya to the Tanzanian border with Mozambique, inland up to an altitude of 1000 m a.s.l. and in the lower altitudes of Malawi (Nichols, 1950). For a long time, CBSD was regarded as a low‐altitude disease generally occurring below 1000 m a.s.l. (Nichols, 1950). Many studies supported this view because the disease was found to be endemic at these altitudes with incidence increasing as altitude decreased (Hillocks *et al*., 2002). Recently, however, CBSD has become more widespread, and its occurrence has been reported at altitudes above 1000 m a.s.l., as in the Democratic Republic of Congo (1231 m a.s.l.; Mulimbi *et al*., 2012), Uganda (1200 m a.s.l.; Alicai *et al*., 2007), western Kenya (>1000 m a.s.l.; Mware *et al*., 2009), northwestern Tanzania (>1000 m a.s.l.; Legg *et al*., 2011; Ndunguru *et al*., 2015) and Burundi (1081–1775 m a.s.l.; Bigirimana *et al*., 2011).

In Rwanda, CBSD‐like symptoms were first reported on the leaves of a few cassava plants in 2009 in the Muhanga district in the south, and in the Bugesera and Nyagatare districts in the east, following a preliminary survey conducted in 14 cassava‐growing districts (Institut des Sciences Agronomiques du Rwanda, unpublished data). Four years later, a survey was conducted in cassava‐growing districts to monitor disease spread. The results from this survey showed that CBSD had spread to new areas such as the Gisagara, Nyanza and Ruhango districts in the southern province and Kirehe district in the eastern province. The 2009 and 2013 surveys reported a national CBSD foliar incidence of 8.8% and 23.3%, respectively (Rwanda Agriculture Board, unpublished data). However, the viruses causing CBSD and their distribution were not determined in either survey. Furthermore, there is lack of information on the symptoms associated with CBSD in Rwanda. The current study therefore aimed to determine the occurrence and distribution of seven CBSD symptom phenotypes in Rwanda and to characterize the associated viruses.

## Materials and methods

### Field assessment of CBSD phenotypes

A field survey was conducted from September to October 2014 in eight major cassava‐growing districts of Rwanda to assess the incidence and severity of seven CBSD phenotypes on leaves, stems and roots, and their combinations, i.e. leaf, stem, root, leaf + stem, leaf + root, stem + root and leaf + stem + root disease symptoms. Districts that showed typical CBSD symptoms in the 2009 and 2013 surveys (Rwanda Agriculture Board, unpublished data) were selected from areas with high levels of cassava production. The districts selected and sampled were Gisagara, Kamonyi, Nyanza and Ruhango in the south and Bugesera, Gatsibo, Kirehe and Nyagatare in the east.

Cassava fields belonging to 160 farmers (20 in each district) were assessed for CBSD. In each district, 10 fields at 6–10 months post‐planting (a stage when CBSD symptoms are clearly visible) were examined for aerial symptoms. Another 10 fields of >10 months post‐planting (when necrosis in storage roots due to CBSD is prominent) were assessed for tuber symptoms. The selected fields were spaced at regular intervals of 5–10 km, although the distance was greater in areas with few cassava fields. Within each field sampled for aerial symptoms, a systematic ‘X’ sampling pattern was used to minimize bias in the estimation of actual disease incidence. A total of 30 plants of a dominant cultivar were assessed for presence and absence of CBSD along two diagonals. The names of other cultivars found in each sampled field were recorded.

The presence or absence of CBSD symptoms on the leaves and stems was recorded for each plant using a scale of 1 to 5 (Gondwe *et al*., 2003). The scale was: 1 = no apparent symptoms; 2 = slight leaf feathery chlorosis with no stem lesions; 3 = pronounced leaf feathery chlorosis, mild stem lesions and no dieback; 4 = severe leaf feathery chlorosis, severe stem lesions and no dieback; and 5 = defoliation, severe stem lesions and dieback. In fields sampled for root symptoms, five plants were uprooted and tuberous roots transversely sliced to examine for root necrosis. Root symptom severity scores were performed using a scale of 1 to 5 as described by Gondwe *et al*. (2003) in which 1 = no apparent necrosis; 2 = <5% root necrosis; 3 = 5–10% root necrosis; 4 = 11–25% root necrosis with mild root constriction; and 5 = >25% root necrosis with severe root constriction.

The incidence of CBSD was calculated from the number of plants exhibiting CBSD symptoms as a percentage of the total number of plants assessed in a field. In calculating mean severity per field, scores of ‘1’ (no visible symptoms) were excluded. This allowed for a true evaluation of the degree of damage caused by CBSD on the affected plants (Sseruwagi *et al*., 2004). Analyses were conducted using spss win v. 12 and GPS readings of altitude, latitude and longitude were recorded for each site.

### Sampling of test materials for PCR

In each field, leaf samples from plants with viral symptoms (where possible) and plants without symptoms were picked for viral testing. Sampling for aerial symptoms included 140 cassava leaf samples from 70 fields on plants showing typical CBSD symptoms on leaves (70), stems (30) and leaves + stems (40). In fields sampled for tuberous root symptoms, a total of 150 cassava leaf samples were collected from 75 fields on plants expressing CBSD symptoms on roots (30), leaves + roots (50), stems + roots (30) and leaves + stems + roots (40). Ninety‐one leaf samples were also collected from apparently CBSD‐free plants. Thus, a total of 381 samples were prepared and tested for the presence of CBSVs using RT‐PCR.

### Extraction of RNA from cassava leaves

Total RNA was extracted from 100 mg of each cassava leaf sample using the cetyltrimethylammonium bromide (CTAB) protocol. The extraction buffer contained 2% CTAB, 1.4 m NaCl, 100 mm Tris‐HCl, 20 mm EDTA, 2% polyvinylpyrrolidone (PVP), and 1% NaSO_3._ Mercaptoethanol was added to the prewarmed extraction buffer (20 μL of mercaptoethanol per mL buffer) before use. The leaf samples were individually ground in a mortar containing 700 μL extraction buffer. Aliquots of 500 μL extract were transferred into 1.5 mL microcentrifuge tubes and incubated at 65 °C for 30 min, while being shaken vigorously every 10 min. The extract was mixed with 700 μL chloroform: isoamyl alcohol (24:1); inverted for 10 min and centrifuged at 16 060 ***g*** for 10 min at 4 °C. The supernatant (500 μL) was transferred into new microcentrifuge tubes to which an equal volume (500 μL) of cold isopropanol was added, and incubated at −20 °C for 30 min. The contents were centrifuged at 16 060 ***g*** for 10 min at 4 °C and the supernatant discarded. The RNA pellet was washed in 500 μL of 70% ethanol, followed by centrifugation at 16 060 ***g*** for 5 min at 4 °C. The ethanol was decanted and the pellet air dried. The dried RNA pellet was resuspended in 50 μL RNase‐free water and treated with DNase (Zymo Research). Yield and purity (A_260_:A_280_ ratio) of the extracted RNA were measured using a NanoDrop 2000 spectrophotometer (Thermo Scientific) and samples with ratios between 2 and 2.4 were used for reverse transcription (RT)‐PCR.

### Virus detection by RT‐PCR

A two‐step RT‐PCR protocol was used for virus detection. Complementary DNA (cDNA) was synthesized from 3 μg total RNA in a 20 μL reaction mixture using NX Gen M‐MuLV reverse transcriptase (Lucigen) primed with oligo(dT)_18_ according to the manufacturer's protocol. The primers CBSDDF2: 5′‐GCTMGAAATGCYGGRTAYACAA‐3′ and CBSDDR: 5′‐GGATATGGAGAAAGRKCTCC‐3′ (Mbanzibwa *et al*., 2011b) were used for PCR amplification of the cDNA template.

The 25‐μL PCR mixture consisted of 2.5 μL 10× reaction buffer, 1 μL 2.5 mm dNTPs, 1 μL 0.4 μm primer, 1 U Dream *Taq* DNA polymerase (Thermo Scientific) and 40 ng cDNA; the reactions were brought to volume with RNase‐free water. Reactions were run in a Gene Amp PCR System 9700 (Applied Biosystems) using the following PCR cycling conditions: denaturation at 94 °C for 2 min; 35 cycles of 94 °C for 30 s, 51 °C for 30 s, 72 °C for 30 s; and a final extension step at 72 °C for 10 min. Products were separated by agarose gel electrophoresis on a 1.5% (w/v) gel in a 1× TAE buffer containing 0.5 μg mL^−1^ ethidium bromide and subsequently viewed under UV light (Biodoc‐It imaging system).

### Nucleotide sequencing and phylogenetic analysis

The presence of cassava brown streak viruses was confirmed by sequencing a number of representative positive samples. Prior to sequencing, PCR‐amplified products were purified using a GeneJET PCR Purification kit (Thermo Scientific) following the manufacturer's instructions. Direct DNA sequencing in both directions was performed at the North Carolina State University Genomic Sciences Laboratory (Raleigh, USA). Sanger cycle sequencing reactions were performed using the BigDye termination mix (Applied Biosystems) and capillary sequencing on an ABI 3730xl DNA Analyzer (Applied Biosystems). Chromatography of sequences was viewed using the ApE program. Sequence quality control through sequence trimming and assembling was performed using CLC genomics workbench software. Quality scores of 0.05 were used for trimming, and sequences with scores rating below 50% were excluded from analyses. Alignment of sequences was performed using muscle in mega v. 7.

Phylogenetic and molecular evolutionary relationships were examined by analysing partial coat protein‐encoding sequences (210 nt) of cassava brown streak viruses. For isolates of CBSV, the sequences started at nucleotide position 8653 to a stop codon with reference to isolate Kor 6 (acc. no. GU563327). For isolates of UCBSV, sequences started at nucleotide position 8841 to a stop codon with reference to isolate UG: Nam 23 (acc. no. FN434109). Analyses were conducted using mega v. 7. The Tamura & Nei substitution model (Tamura *et al*., 2004) was used for maximum likelihood (ML) tree reconstruction. The tree was drawn to scale, with branch lengths measured in the number of substitutions per site. The analyses included 24 UCBSV and six CBSV Rwandan isolates characterized in this study (GenBank accession numbers KX168471 to KX168500) and published CBSV and UCBSV full genome sequences: one isolate of UCBSV (FJ039520) and one isolate of CBSV (NC012698) from Tanzania, three isolates of UCBSV (HG965222, FN434109 and NC014791) from Uganda, two isolates of UCBSV (KR911725, FN433930) and one isolate of CBSV (KR911737) from Kenya, one isolate of UCBSV (FN433933) from Malawi and one isolate of CBSV (FN434436) from Mozambique. The analysis also included *Sweet potato mild mottle virus*, genus *Ipomovirus* and family *Potyviridae* (NC003797) as an out‐group.

To understand the genetic diversity of the isolates, estimates of evolutionary divergence over sequence pairs within and between groups were calculated. The analyses were conducted using the maximum composite likelihood model (Tamura *et al*., 2004). Also, the maximum likelihood estimate of transition/transversion bias (R) was calculated. The substitution pattern and rates were estimated under the Kimura (1980) 2‐parameter model (Tamura *et al*., 2004). Synonymous and nonsynonymous substitution rates influenced by fitness effect were estimated using snap v. 2.1. The analysis was performed on the web‐based platform (http://www.hiv.lanl.gov).

## Results

### Symptoms of CBSD‐affected cassava plants

**Figure 1:**
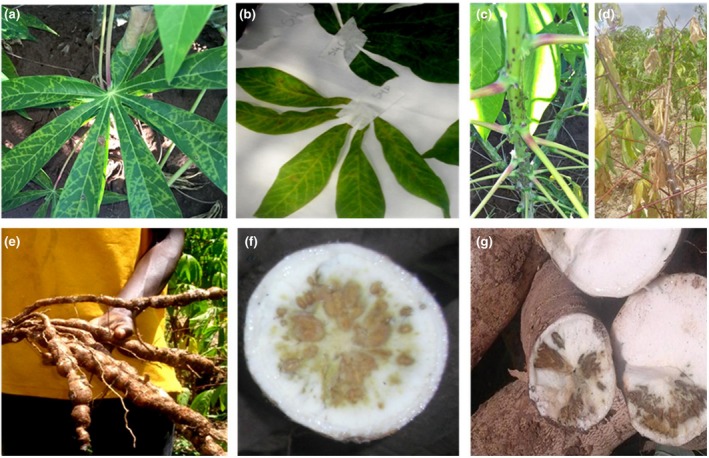
Symptoms of cassava brown streak disease (CBSD) observed on cassava in Rwanda. (a) Interveinal chlorosis and chlorotic blotches. (b) Pronounced yellow mottling and severe vein chlorosis on leaves. (c) Stems of CBSD‐affected cassava plant expressing brown necrosis. (d) Stems of CBSD‐affected cassava plant expressing dieback symptoms. (e) Roots of CBSD‐affected plant showing root constrictions. (f) Roots of CBSD‐affected plant showing uncommon circular yellowish‐brown necrosis. (g) Roots of CBSD‐affected plant showing chalky brown necrosis. [Colour figure can be viewed at wileyonlinelibrary.com]

Variable symptoms were recorded on CBSD‐affected cassava plants in the surveyed fields. Leaf symptoms were yellow patches, chlorotic spots, chlorotic blotches, pronounced mottling and veinal and interveinal chlorosis along the secondary and tertiary veins, which occurred mainly on the lower older leaves (Fig. 1a,b). Stem symptoms manifested as scratch‐like wounds, dark brown spots, and streaks (Fig. 1c). Severe stem systemic necrosis associated with dieback was seen in plants that expressed a combination of stem and leaf symptoms (Fig. 1d). Various levels of root constrictions were recorded, combined with various discolourations (brown, black or yellow, or chalky) in tuberous roots (Fig. 1e,g). There was also evidence of some unique circular symptoms in the roots (Fig. 1f), which are uncommon in CBSD‐affected roots.

### Incidence, severity and distribution of CBSD phenotypes

**Figure 2:**
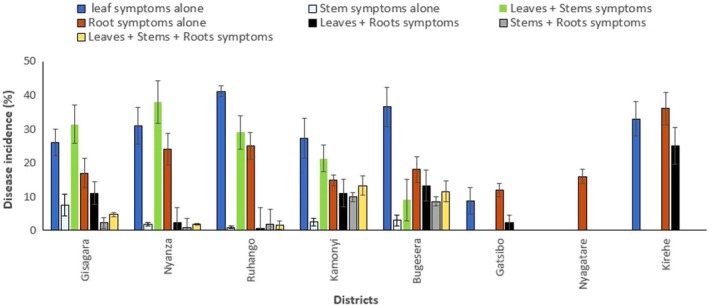
Incidence of cassava brown streak disease (CBSD) phenotypes and their combinations in cassava‐growing districts of Rwanda. [Colour figure can be viewed at wileyonlinelibrary.com]

Cassava brown streak disease phenotypes and their combinations were recorded on the disease‐affected cassava plants. These included: leaves (L), stems (S) and roots (R) alone, and combinations with symptoms on the leaves + stems (L + S), leaves + roots (L + R), stems + roots (S + R) and leaves + stems + roots (L + S + R). There were significant differences (*P *<* *0.001) in the occurrence of the CBSD phenotypes and their combinations in the districts surveyed (Fig. 2). The symptoms on leaf alone had the highest incidence (32.2%). This was followed by root (25.7%), leaf + stem (20.3%), leaf + root (10.4%), leaf + stem + root (5.2%), stem + root (3.7%), and stem (2.5%) symptoms.

Plants expressing CBSD‐root symptoms occurred in all eight districts and the highest (36.0%) and lowest (12.0%) incidences were recorded in Kirehe and Gatsibo districts, respectively. The symptoms on leaf alone occurred in cassava plants examined in all the surveyed districts, except Nyagatare. The highest incidence (41.1%) of symptoms on leaf alone was recorded in Ruhango district. Stem symptoms occurred mainly in the southern districts of Gisagara, Kamonyi Nyanza and Ruhango, while plants with the stem phenotype occurred only in Bugesera district in the eastern region. When considering the combinations of CBSD phenotypes, leaf + root symptoms predominated and recorded 13.3% incidence in Bugesera district. All seven CBSD phenotypes occurred in Bugesera, Gisagara, Kamonyi, Nyanza and Ruhango districts (Fig. 2).

The CSBD phenotypes occurred at different altitudes and in crops of different ages. Leaf, stem and root phenotypes and their combinations occurred mainly at elevations between 1500 and 1700 m a.s.l. The aerial symptoms were clearly present (47.4%) in crops 8–10 months post‐planting. Root symptoms and their combinations were more prominent (60.0%) in crops 15–18 months post‐planting.

Disease severity scores varied little from field to field within a district, cultivar, time since planting, and altitude. The scores ranged from 2.00 ± 0.00 to 2.84 ± 0.04 in leaves, from 2.00 ± 0.80 to 2.40 ± 0.66 in stems and from 2.00 ± 0.00 to 2.86 ± 0.10 in roots (Tables 1 & 2).

**Table 1:** Incidence, severity and frequency of cassava plants showing foliar symptoms of cassava brown streak disease in Rwanda, 2014

	No. of plants	Incidence (%)	Mean severity score	Frequency of severity score (%)				
				1	2	3	4	5
(a) Location (district)								
Southern districts								
Gisagara	300	57.3	2.67 ± 0.06	42.7	28.3	21.3	6.0	1.7
Kamonyi	300	26.3	2.57 ± 0.07	73.7	13.7	10.3	2.3	0.0
Nyanza	300	68.7	2.65 ± 0.05	31.3	33.7	25.7	9.0	0.3
Ruhango	300	90.7	2.84 ± 0.04	9.3	32.7	40.7	16.7	0.6
Eastern districts								
Bugesera	300	64.7	2.52 ± 0.04	35.3	35.3	24.7	4.7	0.0
Gatsibo	300	8.7	2.00 ± 0.00	91.3	8.7	0.0	0.0	0.0
Kirehe	300	61.7	2.29 ± 0.03	38.3	44.3	17.1	0.3	0.0
Nyagatare	300	0.0		100.0	0.0	0.0	0.0	0.0
(b) Cultivar type								
Local	990	25.8	2.47 ± 0.03	74.2	15.3	8.9	1.5	0.1
Improved	1410	62.3	2.63 ± 0.02	37.7	31.1	23.5	7.2	0.5
(c) Crop age (months)								
6–8	540	46.9	2.81 ± 0.05	53.1	19.4	17.0	9.8	0.6
8–10	1860	47.4	2.53 ± 0.02	52.6	26.1	17.6	3.4	0.3
(d) Altitude (m a.s.l.)								
1300–1500	1260	40.4	2.57 ± 0.03	59.6	21.3	15.2	3.6	0.3
1500–1700	1020	56.5	2.64 ± 0.02	43.5	27.6	21.6	6.9	0.4
>1700	120	40.8	2.24 ± 0.07	59.2	32.5	6.7	1.7	0.0

**Table 2:** Incidence, severity and frequency of cassava plants showing root symptoms of cassava brown streak disease in Rwanda, 2014

	No. of plants	Incidence (%)	Mean severity score	Frequency of severity score (%)				
				1	2	3	4	5
(a) Location (district)								
Southern districts								
Gisagara	50	54.0	2.33 ± 0.07	46.0	27.6	20.4	6.0	0.0
Kamonyi	50	28.0	2.50 ± 0.09	72.0	12.0	14.0	2.0	0.0
Nyanza	50	60.0	2.86 ± 0.10	40.0	20.0	30.0	10.0	0.0
Ruhango	50	62.0	2.44 ± 0.07	38.0	28.0	18.0	16.0	0.0
Eastern districts								
Bugesera	50	56.0	2.25 ± 0.07	44.0	28.0	24.0	4.0	0.0
Gatsibo	50	20.0	2.20 ± 0.08	80.0	8.0	12.0	0.0	0.0
Kirehe	50	70.0	2.20 ± 0.06	30.0	46.0	20.0	4.0	0.0
Nyagatare	50	40.0	2.25 ± 0.07	60.0	18.0	14.0	8.0	0.0
(b) Cultivar type								
Local	160	29.4	2.25 ± 0.04	70.6	20.0	9.4	0.0	0.0
Improved	240	61.2	2.42 ± 0.04	38.8	25.8	33.3	2.1	0.0
(c) Crop age (months)								
10–12	285	37.5	2.41 ± 0.04	62.5	25.3	10.5	1.7	0.0
12–15	100	49.0	2.33 ± 0.05	51.0	37.0	12.0	0.0	0.0
15–18	15	60.0	2.00 ± 0.00	40.0	60.0	0.0	0.0	0.0
(d) Altitude (m a.s.l.)								
1300–1500	210	46.2	2.36 ± 0.04	53.8	34.3	11.9	0.0	0.0
1500–1700	175	50.3	2.37 ± 0.05	49.7	33.1	14.3	2.9	0.0
>1700	15	40.0	2.33 ± 0.12	60.0	26.7	13.3	0.0	0.0

There was a considerable variation between cassava cultivars in the expression of the CBSD phenotypes and their combinations. In general, the improved CMD‐resistant cultivars showed more symptoms of CBSD than did local cultivars. Overall, the symptoms on leaves alone were predominant (62.0%) in the improved CMD‐resistant cultivars, whereas in the local cultivars the root symptom phenotype predominated (30.0%). Incidence of symptoms on stems alone was less in both improved (24.1%) and local cultivars (5.9%). When considering the combination of CBSD symptom phenotypes, the occurrence of leaf + root (L+R) symptoms predominated and showed the incidence of 30.8% and 6.9% in improved and local cultivars, respectively (Table 3). All seven phenotypes were observed in 192/0057, 95/NA/00063, MH95/1404, MM96/0287, TME14 and Gapfunsi varieties. Of special note, the cultivar Rutanihisha expressed no CBSD symptoms. Gitamisi and Kwatamumpare expressed root symptoms at a low incidence of 10.0% (Table 3).

**Table 3:** Mean incidence of cassava brown streak disease (CBSD) symptom phenotypes on cultivars commonly produced in Rwanda, 2014

Cultivar	Fields sampled	CBSD incidence (%)						
		Leaves (L)	Stems (S)	Roots (R)	L + S	L + R	S + R	L + S + R
*95/NA/00063*	*16*	*86.0*	*33.0*	*60.0*	*26.3*	*31.1*	*8.9*	*11.1*
Gacyacyari	3	48.4	3.4	20.0	1.7	0.0	0.0	0.0
Gahene	1	13.3	0.0	20.0	0.0	20.0	0.0	0.0
Gapfunsi	2	18.4	15.0	20.0	15.0	13.3	8.7	6.9
Gitamisi	8	44.2	0.0	10.0	0.0	10.0	0.0	0.0
Gumino	2	6.7	0.0	60.0	0.0	20.0	0.0	0.0
*I92/0057*	*40*	*45.9*	*18.7*	*64.2*	*14.1*	*33.7*	*16.8*	*18.9*
Kicaro	14	0.0	0.0	63.3	0.0	0.0	0.0	0.0
Kwatamumpare	13	0.0	0.0	10.0	0.0	0.0	0.0	0.0
*MM96/0287*	*19*	*82.9*	*38.1*	*53.3*	*27.5*	*26.7*	*13.3*	*15.6*
*MM96/3920*	*10*	*82.5*	*0.0*	*83.3*	*0.0*	*0.0*	*0.0*	*0.0*
*MH95/1404*	*5*	*58.4*	*23.4*	*20.0*	*23.4*	*6.7*	*6.7*	*0.0*
Nyiragatuku	1	0.0	0.0	20.0	0.0	0.0	0.0	0.0
*TME 14*	*5*	*21.1*	*3.3*	*70.0*	*1.1*	*40.0*	*0.0*	*0.0*
Rutanihisha	3	0.0	0.0	0.0	0.0	0.0	0.0	0.0
Unnamed^a^	18	54.3	15.7	37.5	8.7	15.0	5.0	5.0
All local (*n *=* *9)	47	25.9	5.9	30.0	3.6	6.9	1.3	1.3
All improved (*n *=* *6)	*95*	*62.0*	*24.1*	*61.3*	*17.1*	*30.8*	*11.3*	*12.5*
All cultivars (*n *=* *15)	160	47.2	16.6	48.8	11.5	21.3	7.3	8.0

All improved cassava mosaic disease (CMD)‐resistant cultivars and corresponding statistics are indicated in italics.

a Cultivars for which farmers had no names.

Some varieties showed differences in symptom expression across the different geographical districts surveyed. Kizere variety expressed leaf, stem and root symptom phenotypes and their combinations in the districts of Gisagara, Nyanza, Ruhango and Kamonyi. However, this variety did not show stem symptoms or its combinations in Bugesera and Gatsibo districts (data not shown).

### Viruses associated with CBSD phenotypes

**Figure 3:**
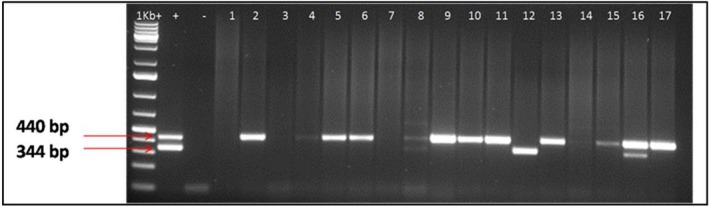
Detection of *Cassava brown streak virus* (CBSV) and *Ugandan cassava brown streak virus* (UCBSV) by reverse transcription‐PCR in samples collected from the field using primer pairs CBSDDF2 and CBSDDR. Expected product sizes were: CBSV (344 bp) and UCBSV (440 bp). 1 kb +, DNA ladder; +, positive control for both CBSV and UCBSV; −, negative control; lane 1, leaf sample from Nyagatare; lane 2, Gatsibo; lane 3, Kirehe; lane 4 and 5, Bugesera; lane 6 and 7, Rusizi; lane 8, 9 and 10, Nyanza; lane 11 and 12, Kamonyi; lane 13 and 14, Gisagara; lane 15, 16 and 17, Ruhango. [Colour figure can be viewed at wileyonlinelibrary.com]

**Figure 4:**
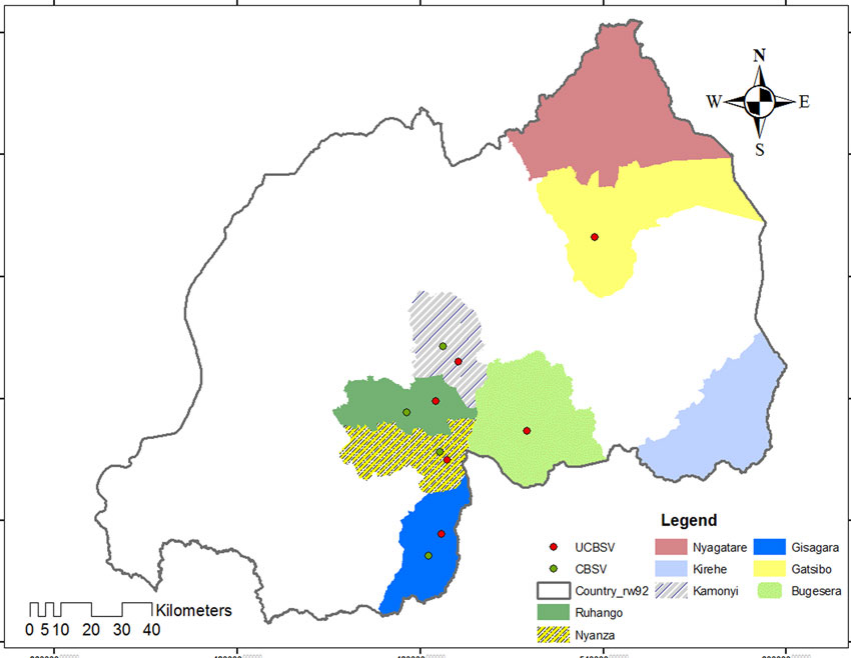
Distribution of *Cassava brown streak virus* (CBSV) and *Ugandan cassava brown streak virus* (UCBSV) in the eight districts of Rwanda surveyed, 2014. [Colour figure can be viewed at wileyonlinelibrary.com]

The viruses associated with the different CBSD phenotypes were investigated by RT‐PCR using virus‐specific primers and the analyses included some symptomless plants (Table 4a). Two virus species, CBSV and UCBSV, were detected in the samples as single (CBSV or UCBSV) or mixed infections (CBSV + UCBSV; Fig. 3). Single infections of UCBSV were detected in six of the eight districts surveyed, in Kamonyi, Gisagara, Nyanza and Ruhango (southern) and Bugesera and Gatsibo (eastern). CBSV occurred in all four southern districts; however, it was not detected in the eastern districts (Fig. 4). Mixed infections occurred only in the Nyanza and Ruhango districts of the south. No cassava brown streak viruses were detected in the districts of Kirehe and Nyagatare (Table 4b).

**Table 4:** Incidence of cassava brown streak viruses in cassava brown streak disease (CBSD)‐affected plants expressing various symptoms and sampled from different cassava‐growing districts of Rwanda

	Leaf samples tested	CBSV	UCBSV	CBSV + UCBSV	Total infection
(a) Symptom types					
Leaf	70	6 (8.6)	31 (44.3)	6 (8.6)	43 (61.4)
Stem	30	5 (16.7)	11 (36.7)	0 (0.0)	16 (53.3)
Root	30	0 (0.0)	2 (6.7)	0 (0.0)	2 (6.7)
Leaf + stem	40	5 (12.5)	30 (75)	0 (0.0)	35 (87.5)
Leaf + root	50	3 (6.0)	11 (22.0)	5 (10.0)	19 (38.0)
Stem + root	30	0 (0.0)	19 (63.3)	0 (0.0)	19 (63.3)
Leaf + stem + root	40	6 (15.0)	17 (42.5)	6 (15.0)	29 (72.5)
Total with symptoms	290	25 (8.6)	121 (41.7)	17 (5.9)	163 (56.2)
Symptomless	91	1 (1.1)	11 (12.1)	0 (0.0)	12 (13.2)
All total	381	26 (6.8)	132 (34.6)	17 (4.5)	175 (45.9)
(b) District					
Bugesera	40	0 (0.0)	27 (67.5)	0 (0.0)	27 (67.5)
Gatsibo	40	0 (0.0)	11 (12.5)	0 (0.0)	11 (12.5)
Gisagara	40	4 (10.0)	27 (67.5)	0 (0.0)	31 (77.5)
Kamonyi	30	4 (13.3)	17 (56.7)	0 (0.0)	21 (70.0)
Kirehe	40	0 (0.0)	0 (0.0)	0 (0.0)	0 (0.0)
Nyagatare	20	0 (0.0)	0 (0.0)	0 (0.0)	0 (0.0)
Nyanza	40	8 (20.0)	22 (55.0)	5 (12.5)	35 (87.5)
Ruhango	40	9 (22.5)	17 (42.5)	12 (30.0)	38 (95.0)
Total	290	25 (8.6)	121 (41.7)	17 (5.9)	163 (56.2)

Figures in parentheses are percentage values. CBSV, *Cassava brown streak virus*; UCBSV, *Ugandan cassava brown streak virus*.

Single infections of UCBSV were detected in several cassava plants, irrespective of the CBSD phenotype. However, single UCBSV infections predominated in plants with the leaf + stem phenotype at an incidence of 75.0%. UCBSV occurred in only 6.7% of CBSD‐affected plants expressing the root phenotype. Single infections of CBSV were predominant in CBSD‐affected plants showing stem symptoms although at a low incidence of only 16.7%. CBSV did not occur in plants expressing CBSD‐root symptoms alone. The highest (15.0%) incidence of co‐infection (CBSV + UCBSV) was recorded in plants with the CBSD leaf + stem + root phenotype. Interestingly, UCBSV was detected in 11 of the 12 CBSD‐symptomless plants (Table 4a).

### Molecular diversity of viruses associated with CBSD phenotypes

**Figure 5:**
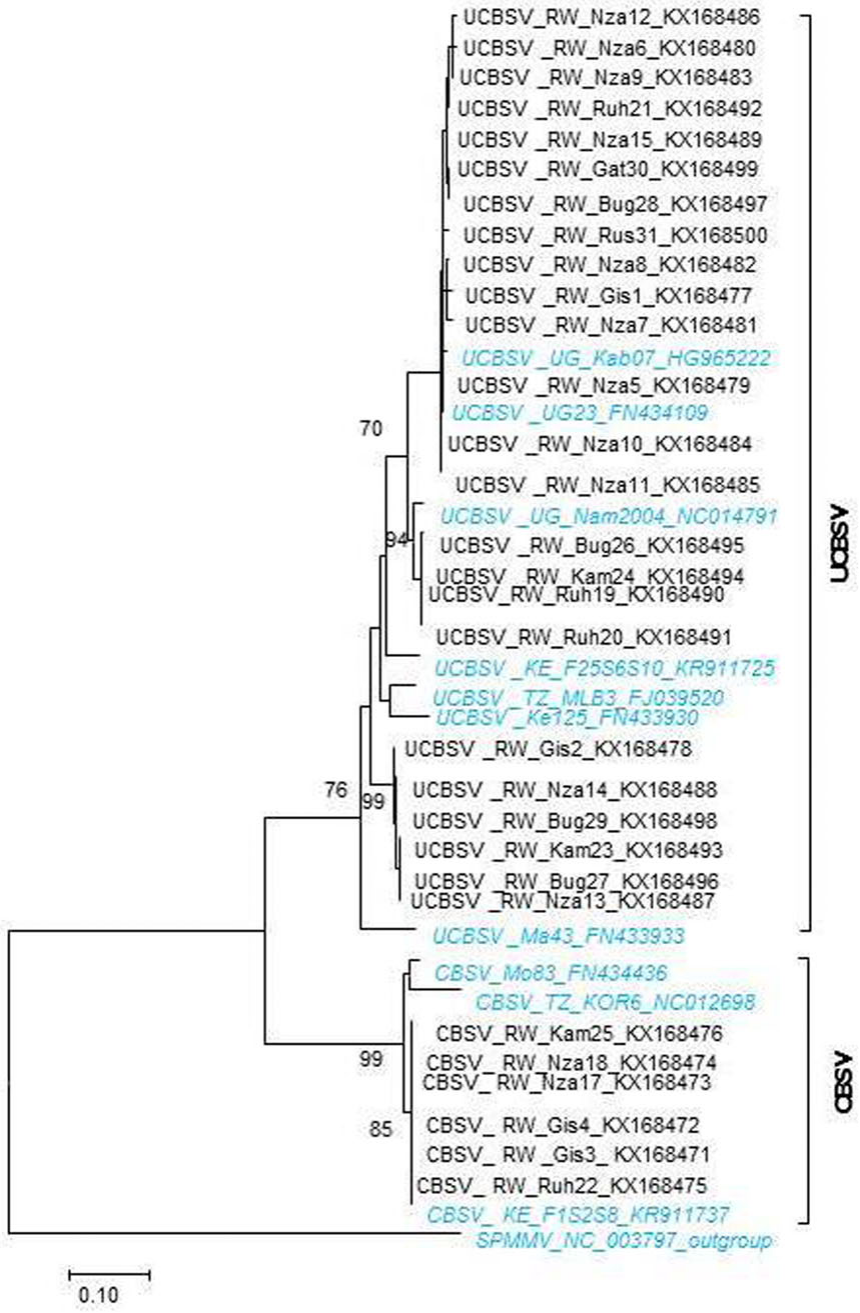
Molecular phylogenetic analysis by maximum likelihood method of partial coat protein‐encoding sequences (210 nt) of cassava brown streak viruses. The analysis included isolates of *Cassava brown streak virus* (CBSV) and *Ugandan cassava brown streak virus* (UCBSV) characterized in this study (from Rwanda, RW; in black) and isolates characterized previously (in italic, blue). In addition, a *Sweet potato mild mottle virus* (SPMMV) isolate was included in the analysis as an out‐group. The evolutionary history was inferred by using the maximum likelihood based on the Tamura & Nei method (Tamura *et al*., 2004). The tree is drawn to scale of 0.1, with branch lengths measured in the number of substitutions per site. Evolutionary analyses were conducted in mega 7 (Kumar *et al*., 2016). [Colour figure can be viewed at wileyonlinelibrary.com]

Analysis of 30 partial coat protein (CP) gene sequences (210 nt) confirmed the occurrence of both CBSV and UCBSV in Rwanda (Fig. 5). Six isolates from Gisagara (2), Kamonyi (1), Nyanza (2) and Ruhango (1) districts clustered together in the CBSV clade. The CBSV isolates identified in this study formed only one group and they were very close if not identical to the isolate KR911737 from Kenya. Another 24 isolates clustered with the UCBSV clade and were identified in Gisagara (2), Kamonyi (2), Nyanza (11), Ruhango (3), Bugesera (4) and Gatsibo (2) (Fig. 5). The UCBSV isolates formed three groups. The first and second groups comprised the isolates that are highly related to Ugandan isolates HG965222 and NC014791, respectively. The third group that comprised six isolates, two from Nyanza district (KX168487, KX168488), one from Gisagara (KX168478), one from Kamonyi (KX168493) and two from Bugesera (KX168496, KX168498), was well separated from the other two groups and these isolates were distantly related to those previously published.

The estimates of average evolutionary divergence over sequence pairs within groups showed that the number of base substitutions per site, from averaging over all sequence pairs within UCBSV and CBSV groups, was 0.072 and 0.025, respectively. The number of base substitutions per site between UCBSV and CBSV was 0.36. The estimated transition/transversion bias (R) for UCBSV and CBSV was 7.26 and 6.08, respectively. The number of synonymous substitutions (dS) and nonsynonymous substitutions (dN) per site was found to be zero in CBSV. In UCBSV, dS was 0.24, dN was 0.12 and the ratio of synonymous to nonsynonymous (dS/dN) was 3.49.

## Discussion

This paper reports, for the first time, the occurrence of both CBSV and UCBSV in Rwanda, based on results obtained from partial sequencing of the CP gene. There are unpublished reports of only UCBSV in the country in the districts of Bugesera, Kamonyi and Ruhango. However, this covers only limited areas and it is likely that the few samples studied at that time were not representative of the full area affected by CBSD in Rwanda.

The current study established the occurrence and distribution of CBSD‐associated phenotypes and the associated viruses through extensive collections of CBSD‐affected leaf samples in eight cassava‐producing districts in southern and eastern Rwanda. It was found that UCBSV was more widespread than CBSV; interestingly, CBSV occurred only in the southern districts. These observations indicate that UCBSV is the most important virus infecting cassava in Rwanda. Generally, the symptoms recorded on the CBSD‐affected plants were typical, except for a few roots that had unique circular yellowish‐brown symptoms. The most common root symptom is reported to be necrosis (Hillocks & Jennings, 2003), and circular yellowish‐brown symptoms have been rarely reported in the literature. The cause of this symptom was not clear and it is suggested that future studies should investigate the cause and genes involved in its expression.

With no previous occurrence of CBSD in Rwanda, it is likely that the disease was imported from neighbouring countries, possibly with untested cassava germplasm. This seems probable because, in the present study, the isolates of viruses characterized were closely related to isolates from East African countries (Uganda, Tanzania and Kenya). Furthermore, CBSD was mainly observed in the improved CMD‐resistant cultivars introduced from neighbouring Uganda that were subsequently propagated and distributed to farmers in Rwanda. This was done with a view to controlling the CMD pandemic into Rwanda two decades ago (Night *et al*., 2011). The improved materials may not have been CBSD‐free and neither were they CBSD‐resistant before being introduced, as at that time CBSD was not considered a problem.

Elsewhere (in Uganda and western Kenya), the elite CMD‐resistant varieties that championed the management of the severe CMD pandemic were reported to be highly susceptible to CBSD (Ntawuruhunga & Legg, 2007; Mware *et al*., 2009). This has hugely undermined the efforts by breeders and pathologists to control cassava viruses, posing an enormous challenge to ensuring food security for cassava‐dependent rural households. Efforts have been made in Tanzania, Uganda and Kenya to develop CBSD‐resistant cassava varieties and to manage the problem of CMD‐resistant but CBSD‐susceptible genotypes by searching for dual resistance/tolerance. The efforts were partially successful and further contributions to broaden the genetic base of CBSD resistance would result in resistance diversification and thus reduce the disease epidemics. In the present study, some local varieties, notably Rutanihisha and Kwatamumpare were less affected or unaffected by CBSD symptoms. Considering that varieties tolerant to CBSD may express mild brown streaks in the roots, while the stems and leaves remain symptomless (Jennings, 1960), the present data suggest Rutanihisha and Kwatamumpare varieties as candidates for CBSD‐tolerance. However, this needs to be verified by screening Rwandan germplasm to identify and characterize sources of resistance to CBSD in these varieties and other local Rwandan cassava varieties.

In the current study, considerable variation was found in the occurrence and distribution of CBSD phenotypes on cassava in Rwanda. Leaf and root phenotypes were the most dominant, with 32.25% and 25.7% incidence, respectively. Of the combined CBSD phenotypes, leaf + stem symptoms was the most prominent (20.3%). This study reports the spread of CBSD in new cassava‐growing districts, such as Gatsibo in the east, where the disease was not reported in the 2009 and 2013 surveys (Rwanda Agriculture Board, unpublished data). In addition, an increase of foliar CBSD incidence from 8.8% in 2009 and 23.3% in 2013 to 47.2% in 2014 has been reported. The rapid spread and increase of CBSD incidence observed over the 5 years since CBSD‐like symptoms were first observed in Rwanda could be associated with the exchange and propagation of CBSD‐affected cuttings among farmers. There is a general lack of knowledge of CBSD, its cause, how it spreads and the associated symptoms among cassava farmers. Therefore, it is suggested that great care must be taken when distributing planting material and the viral status of cuttings should be checked before introduction.

Analysis of the association between virus and CBSD phenotype showed that single infections of UCBSV were detected in several cassava plants irrespective of the CBSD phenotype. However, single infections of CBSV were predominant in CBSD‐affected plants showing stem symptoms, although at a low incidence. Single infections of CBSV did not occur in plants expressing CBSD root symptoms alone. In a study by Moreno *et al*. (2011), symptom expression has been shown to correlate with virus load in different organs. This suggests that, in the present study, CBSV replicated more highly in stems than in other parts of cassava crops. However, in the present study, leaf samples were used for the molecular detection of viruses (the most common type of sample used for identifying viruses in cassava and many other crops); therefore, as roots were untested, it is unknown whether CBSV was able to colonize root tissues in some varieties. Nevertheless, in other studies, it has been reported that some mostly tolerant genotypes accumulated a higher titre of CBSV in storage roots than in aerial organs (Ogwok *et al*., 2015). The present results emphasize the importance of the choice of sample to be used for reliable diagnostics and stress the need for the incorporation of stems and roots as samples for routine CBSV molecular detection. In addition, studies on replication and distribution of virus in infected tissues would improve understanding of the relationship between the occurrence of virus and symptom expression.

Variation in phenotype was observed in Kizere variety in different districts surveyed. This variety showed stem symptoms in southern districts but not in eastern districts, which could be attributed to effects of the environment; it has been reported that all parts of CBSD‐affected plant can exhibit symptoms, but the phenotype manifested depends on different factors such as environmental conditions and varietal sensitivity (Hillocks & Jennings, 2003). However, the disparity in distribution of phenotypes in the districts surveyed was due largely to the virus profile because the absence of stem symptoms on Kizere was noticed in districts where CBSV was not detected. No specific difference in symptoms was observed between CBSV and UCBSV, which is consistent with previous reports (Legg *et al*., 2015; Ndunguru *et al*., 2015; Ogwok *et al*., 2015).

A combination of symptomatology and molecular detection of CBSD‐causative viruses gives a better diagnosis. The nondetection of virus in samples with symptoms tested in this study, especially samples collected from Nyagatare and Kirehe, could be explained by the occurrence of new variants of viruses that cannot be detected by the current diagnostic primers. Indeed, it is reported that there might be more than two species of virus (Ndunguru *et al*., 2015; Alicai *et al*., 2016). From the current study, there was no evidence for nondetection of viruses in samples collected from Kirehe and Nyagatare districts of the eastern province. Deep sequencing of the total RNAs of viruses in samples from these districts and the rest of Rwanda could provide valuable additional information.

In the current study, partial CP gene sequences were used to characterize the cassava brown streak viruses occurring in Rwanda. The phylogenetic relationship analyses showed that CBSV isolates in Rwanda are identical. However, there was an indication of diverse UCBSV in the country. When the genetic diversity based on the estimates of evolutionary divergence and transition/transversion bias were examined, it was found that CBSV and UCBSV have different evolutionary patterns, although with less substitutions per site. The results suggest that, in Rwanda, there is one type of CBSV and genetic diversity in UCBSV species exists. The values obtained from the ratio of synonymous to nonsynonymous substitutions showed that the viruses are passing through positive selection. However, the short length of sequences used in this study provides limited evidence for definitive conclusions on selection pressure of CBSV and UCBSV. When the isolates characterized in this study were compared to the ones previously published, it was found that they were closely related to the isolates from neighbouring countries except for a group of six isolates that were well separated from other UCBSV isolates. From this study, it may be speculated that these six isolates originated from Rwanda, but further studies on genetic diversity of cassava brown streak viruses in Rwanda based on full genome sequences would give better insight.

The data from this study highlight the threat that CBSD poses to sustainable cassava productivity in Rwanda, and its impact on food security and income of smallholder farmers. The description of symptoms associated with cassava brown streak viruses may be useful for diagnosis of CBSD. The data will contribute to enhanced integrated disease management in cassava crops. To reduce both disease incidence and the risk of more virulent isolates emerging, implementation of effective CBSD surveillance, promotion and strengthening of phytosanitation measures and quarantine systems is required within the country and at the regional level.
